# Characteristics of Occupational Therapy Interventions to Promote Healthy Aging: Protocol for a Scoping Review

**DOI:** 10.2196/55198

**Published:** 2024-03-18

**Authors:** Katrina Bannigan, Nicole Jade Larkan, Emmanuelle Renee Rogelia Meurgue, Jason Chun Hin Sze

**Affiliations:** 1 School of Health and Life Sciences Glasgow Caledonian University Glasgow United Kingdom

**Keywords:** healthy aging, interventions, occupational therapy, public health, scoping review, intervention, aging, lifestyle change, aging population, well-being, occupation-based approach, occupational therapists, older people

## Abstract

**Background:**

Healthy aging is a pressing public health priority. Focusing on what people do every day may be a meaningful approach to lifestyle change, suggesting a need for occupation therapy interventions to promote healthy aging. A preliminary database search was conducted, and no current or underway systematic or scoping reviews on the topic were identified. Developing an overview of studies of occupational therapy interventions to promote healthy aging is a necessary first step to understanding the existing knowledge and increasing the impact of future interventions. This scoping review will build on previously conducted reviews.

**Objective:**

This scoping review will identify the following: (1) what occupational therapy interventions exist for promoting healthy aging in community-dwelling adults? and (2) what are the intervention characteristics, their evaluated outcome, and the impact observed?

**Methods:**

This protocol was reviewed by 2 occupational therapists as part of a patient and public involvement consultation. The review will consider all studies and publications of occupational therapy focused on promoting healthy aging in community-dwelling adults who are aged 18 years and older. Databases to be searched are AMED, CINAHL, Cochrane Library, Embase, JBI EBP database, MEDLINE, OAlster, PsycINFO, PsycArticles, ProQuest Dissertations & Theses, ProQuest nursing and allied health source, PubMed, and Science Direct. Studies published in any language will be included. Titles and abstracts will be screened against the inclusion criteria using Covidence (Veritas Health Innovation). Potentially relevant studies will be retrieved in full and assessed against the inclusion criteria. No date limiters will be used. Study selection will be completed by 2 independent reviewers. Data will be extracted using a data extraction tool, including descriptive characteristics of the participants including age, sex, and socioeconomic status. Data will be charted using the TIDieR (Template for Intervention Description and Replication) checklist in alignment with the review objectives. The scoping review will be reported in accordance with the PRISMA-ScR (Preferred Reporting Items for Systematic Reviews and Meta-Analyses Extension for Scoping Reviews) statement.

**Results:**

The research began in October 2023, and the results are expected to be published in 2024.

**Conclusions:**

This scoping review will produce valuable information about occupation-based interventions to promote healthy aging to support the development of an occupational therapy intervention.

**Trial Registration:**

Open Science Framework 5k36d; https://osf.io/5k36d/

**International Registered Report Identifier (IRRID):**

DERR1-10.2196/55198

## Introduction

### Overview

The proportion of older people in society will double to 22% between 2015 and 2050 [1]. To this end, the United Nations has instituted a *decade of healthy aging 2021-2030* aimed at promoting healthy aging and addressing the challenges associated with an aging population across the life course [2]. This means active aging and healthy lifestyles have become priorities for public health internationally [3]. Healthy aging is “the process of developing and maintaining the functional ability that enables well-being in older age” [4]. It comprises the intrinsic capacity of the individual—their physical and mental capacities, environmental characteristics, and the interactions between them [2]. It is not necessary to be completely free from illness or disability to achieve healthy aging [2]. A meaningful life, such as a life filled with worthwhile activities [5] or being able to remain living in the community, may help promote successful and healthy aging [6]. This suggests an occupation-based approach centered on what people do every day could be a key to unlocking the 5 pillars to aging well, namely, healthy eating, hydration, exercise, social connections, and cognitive health [7]. Occupational therapists provide opportunities for individuals to engage in activities and live how they wish at every stage of life, and this may offer a meaningful approach to healthy aging [8]. It has been suggested that “The occupational therapy profession has an essential role to play in healthy aging that includes enabling participation” [9].

Occupational therapists already work in this field and occupation-based health promotion interventions exist [10]. However, some of the existing reviews are no longer current; for example, Gustafsson et al [11], Arbesman and Mosley [12], and Stav et al [13] have not been updated. A review of older people’s lived experience of occupation and well-being, based on 3 studies of moderate quality, suggests there is a relationship between well-being and occupation for older people. This is shaped by 2 factors: “first, variation and independence in undertaking activities; second, having a choice between the occupations and a structure of activities that make up daily life. The two factors are influenced by a balance between having activities alone and with others.” [14]. A review of economic evaluations of occupational therapy for people with cognitive or functional decline, or both, found that evidence was scarce, with few comprehensive high-quality economic evaluations having been conducted, but those that have been conducted suggest the interventions are effective and have economic benefits [15].

The literature focuses on older people, rather than other points in the life span, despite the life course approach endorsed by the United Nations’ *decade of healthy aging 2021-2030* [2]. Nonetheless, the population of older adults has been variously defined as 60 years old [13], over 65 years of age or older [9], or 80 years of age or older [12]. Interventions have targeted people living in nursing homes [16], Australian residential aged care facilities [17], and community-dwelling older people [10,17,18]. The focus has tended to be on secondary prevention, within the context of disease management, rather than primary prevention with community-dwelling adults [10]. No study was identified that delineated the characteristics of the interventions reviewed, which is a necessary first step to understanding the existing knowledge and supporting the design, evaluation, and impact of future interventions [19]. Intervention development should be explicit about the content of the intervention, its theoretical foundation, and the context in which the intervention is delivered [11]. Therefore, to further understand how occupational therapy can contribute to promoting healthy aging, it is important to understand the interventions available, their characteristics, and the impact observed [20].

A scoping review is a first step toward identifying what occupational therapy interventions exist to promote healthy aging for community-dwelling adults. A preliminary search of Cochrane Library, figshare, JBI Evidence Synthesis MEDLINE, Open Science Framework, PROSPERO, and PubMed was conducted. The search identified 11 recent reviews. While the reviews identified had a bearing on the topic, most were not specifically about the topic. A total of 7 of the 11 reviews were about discrete topics, that is, older adults’ occupations in heatwaves [21], joy among older people [22], quality of life [16,23], e-tools for transportation planning [24], occupational therapy in residential aged care [17], and instruments to support occupational therapy practice [9]. A review by Kim et al [18] focused on daily functioning and included occupational therapy, but its focus was on activities of daily living and instrumental activities of daily living rather than the broader concept of healthy aging. A scoping review by Mehrotra et al [20] about healthy aging and occupational therapy in South Asian countries was potentially replicable for a wider population, but the full report is not available and the authors of the protocol have not responded to requests for the full report. The rapid review by Owusu-Addo et al [25] was about implementation approaches rather than interventions. Occupational therapy was included in the review of the theory-based specification of nonpharmacological treatments in aging and dementia by Sikkes et al [26], but it did not cover specific interventions and its focus was dementia—secondary prevention—rather than healthy aging more generally. This means that no existing or contemporary review was identified, but these reviews provide valuable insights into how to conduct a review in this field. For example, keywords for searching should include productive aging [10], aging well, healthy aging, positive aging, and successful aging [20]. Thus, a scoping review will be conducted to provide an overview of occupational therapy in relation to promoting healthy aging and discover gaps in knowledge. This will provide up-to-date evidence about occupational therapy interventions to promote healthy aging and its characteristics [19].

### Review Questions

This scoping review will identify the following: (1) what occupational therapy interventions exist for promoting healthy aging in community-dwelling adults? and (2) what are the intervention characteristics, their evaluated outcome, and the impact observed?

## Methods

### Study Design

The proposed scoping review will be conducted in accordance with the Joanna Briggs Institute (JBI) methodology for scoping reviews [27]. This scoping review protocol is registered on the Open Science Framework [28] and will be reported in accordance with the PRISMA-P (Preferred Reporting Items for Systematic Review and Meta-Analysis Protocols) statement [29]. This scoping review protocol will be conducted using the JBI guidelines for scoping reviews to ensure a systematic methodology that can be replicated and reported in line with the PRISMA-ScR (Preferred Reporting Items for Systematic Reviews and Meta-Analyses Extension for Scoping Reviews) [30].

### Eligibility Criteria

#### Participants

This review will consider studies of occupational therapy focused on promoting healthy aging in adults living in the community—community-dwelling—aged 18 years or older. This may include adults living in their own homes or a care setting. Studies will be excluded if they focus on children.

#### Concept

The concept being mapped in this scoping review will be occupational therapy interventions to promote healthy aging in the context of the life course approach. The life course approach acknowledges that health and well-being are influenced by social, economic, and environmental factors throughout a person’s life, as well as personal characteristics [31]. The terms active aging and healthy aging, although subtlety different, are often used interchangeably, that is, active aging refers to “optimizing opportunities for health, participation and security in order to enhance the quality of life as people age” [32], and healthy aging is defined as “developing and maintaining the functional ability that enables well-being in older age” [4], where functional ability includes people’s ability to meet their basic needs to ensure an adequate standard of living, learning, growing and making decisions, being mobile, building and maintaining relationships, and contributing to society [2]. Therefore, this scoping review will consider studies with interventions that focus on active aging or healthy aging as part of health promotion, that is, primary prevention. Studies will be excluded if they address secondary prevention, that is, interventions that focus on the early stages of disease, such as fall prevention for older adults following a fall, or tertiary prevention, that is, interventions during the symptomatic phase of a disease to minimize disability and maximize quality of life, such as foot care for people living with diabetes.

The word “occupation,” in the context of occupational therapy, refers to the everyday activities that humans engage in as part of their daily routine to occupy time, providing purpose and meaning to their lives [33,34]. Occupations are the activities people want to do, need to do, or are expected to do [34]. Examples of these meaningful activities can include social interaction with peers, cooking, different forms of exercise, and working [35]. Occupation-based interventions aim to enhance the performance of an individual when undertaking meaningful activities of daily living by increasing their social and community interaction [36]. Occupation-focused interventions encapsulate the rapid improvement of an individual’s performance through compensatory methods by implementing adaptive measures to be tailored to their current performance level [37]. Interventions are implemented to increase the performance of the individual in the desired or necessary tasks [38].

#### Context

This review will consider studies from any setting, in any country. As a global perspective will be considered, it means no limit on language will be made in the search strategy. This will ensure valuable insights are not lost because they were published in a language other than English [39]. As no language limits will be imposed, relevant papers that are not published in the English language will be translated using Google Translate. Jackson et al [40] have revisited the work by Balk et al [41], who recommended that Google Translate can be used with caution because Google has updated its translation engine. Jackson et al [40] concluded that it is a viable, accurate tool for translating non-English language trials for the purpose of conducting systematic reviews, which is why we will use it for our review. We appreciate that a scoping review is more narrative based than a systematic review; therefore, if we have access to a native speaker, the translation will be checked by them. If it is not possible to verify the translation, this will be reported within the review report. The number and sources of non-English language literature will be reported in the review report.

### Types of Sources

This scoping review will consider both experimental and quasi-experimental study designs, including randomized controlled trials, nonrandomized controlled trials, before and after studies, and interrupted time-series studies. In addition, analytical observational studies, including prospective and retrospective cohort studies, case-control studies, and analytical cross-sectional studies will be considered for inclusion. This review will also consider descriptive observational study designs, including case studies, individual case reports, and descriptive cross-sectional studies for inclusion. Qualitative studies will also be considered that focus on qualitative data including, but not limited to, designs such as phenomenology, grounded theory, ethnography, qualitative description, action research, and feminist research. In addition, systematic reviews that meet the inclusion criteria will also be considered depending on the research question. Text and opinion papers will also be considered for inclusion in this scoping review.

### Review Team

The review is being conducted by a team comprised of an academic (KB) and 3 postgraduate researchers (JCHS, NJL, and ERRM).

### Patient and Public Involvement

The scoping review protocol has been reviewed by 2 occupational therapists working in the field of healthy aging or with older people.

### Search Strategy

The search strategy will aim to locate both published and unpublished studies. An initial limited search of AMED, CINAHL, Cochrane Library, MEDLINE PsycINFO, and PubMed was undertaken to identify if a review of this topic has previously been conducted. The text words contained in the titles and abstracts of relevant studies, and the index terms used to describe the studies were used to develop a full search strategy for AMED, CINAHL, Cochrane Library, Embase, JBI EBP database, MEDLINE, OAlster, PsycINFO, PsycArticles, ProQuest nursing and allied health source, ProQuest Dissertations & Theses, PubMed, and Science Direct (see Multimedia Appendix 1 for an example search strategy). The search strategy, including all identified keywords and index terms, will be adapted for each included database and information source. The reference list of all included sources of evidence will be screened for additional studies. The searches will be conducted by KB, NJL, JCHS, and ERRM. No date or language limiters will be used.

### Study or Source of Evidence Selection

Following the search, all identified citations will be collated and uploaded into Covidence [42] and duplicates will be removed. Following a pilot test, titles and abstracts will then be screened by 2 or more independent reviewers for assessment against the inclusion criteria for the review. Potentially relevant sources will be retrieved in full, and their citation details imported into Covidence [42]. The full text of selected citations will be assessed in detail against the inclusion criteria by 2 or more independent reviewers. Reasons for the exclusion of sources of evidence that do not meet the inclusion criteria during full-text screening will be recorded and reported in the scoping review. Any disagreements that arise between the reviewers at each stage of the selection process will be resolved through discussion or with additional reviewers. The results of the search and the study inclusion process will be reported in full in the final scoping review and presented in a PRISMA-ScR flow diagram [30].

### Data Extraction

A draft extraction form is provided (see Multimedia Appendix 2). The draft data extraction tool may be modified and revised as necessary during the process of extracting data from each included evidence source. Modifications will be detailed in the scoping review. Any disagreements that arise between the reviewers will be resolved through discussion or with additional reviewers. If appropriate, authors of papers will be contacted to request missing or additional data, where required.

### Ethical Considerations

As this scoping review covers reviewing and extracting data from publicly available materials, this study does not require ethics approval.

### Data Analysis

The TIDieR (Template for Intervention Description and Replication) checklist will be used in this review to ensure completeness in the reporting of the characteristics of interventions [43]. The extracted data will be charted in tabular form using the TIDieR checklist. The charted results will be accompanied by a narrative summary and will describe the relationship between the result and the review’s objectives and questions. The results will be presented in accordance with the TIDieR format and the main conceptual categories used in the extraction too, as well as gaps in the existing literature. These results will be presented in relation to the question of this scoping review. The narrative will be guided by the Patterns, Advances, Gaps, Evidence for practice and Research recommendations (PAGER) framework [44]—a structured approach to the analysis and reporting of scoping reviews.

## Results

The research began in October 2023, and the results are expected to be published in 2024.

## Discussion

### Findings

This scoping review will produce a map of the current body of research about occupational therapy interventions to promote healthy aging among community-dwelling adults. As far as we know, this is the first scoping review that maps the occupational therapy interventions that exist to promote healthy aging for community-dwelling adults, the intervention characteristics, and their evaluated outcome and impact that is not limited to any country. Similar to any review, there exist limitations when conducting a scoping review. Although a rigorous identification and inclusion strategy has been delineated, there is still a risk that some valuable information, which could have enhanced our comprehension, might be inadvertently excluded. This is particularly prevalent in the case of grey literature, which may not always come up in academic searches. As the aim and nature of a scoping review are to explore the breadth of a topic to see what has been done, but not the depth, the reviewers are not able to evaluate the quality of the studies that are incorporated within the scoping review. Although scoping reviews can be helpful in generating hypotheses and identifying research gaps, they may not offer the same degree of thoroughness or meticulousness as a systematic review that involves a more rigorous and comprehensive methodology for data collection and analysis and, consequently, may offer a higher level of evidence than scoping reviews [45]. A methodological appraisal of the included studies will not be performed. It is important to note that the limitations and results presented in the suggested review may not be all-encompassing or investigated beyond the peer-review stage of the published paper.

### Conclusions

The review authors will use the findings of this scoping review to support the development of a manualized occupational therapy intervention [46] to promote healthy aging within the EmpowerAge program being developed at Glasgow Caledonian University.

## Appendix

Multimedia Appendix 1 Search strategy.

Multimedia Appendix 2 Data extraction instrument.

## Abbreviations

JBI: Joanna Briggs Institute
PAGER: Patterns, Advances, Gaps, Evidence for practice and Research recommendations
PRISMA-P: Preferred Reporting Items for Systematic Review and Meta-Analysis Protocols
PRISMA-ScR: Preferred Reporting Items for Systematic Reviews and Meta-Analyses Extension for Scoping Reviews
TIDieR: Template for Intervention Description and Replication

## Data Availability

Data sharing is not applicable to this study as no data sets were generated or analyzed during protocol development.
