# Supplementary material for: Characteristics of Occupational Therapy Interventions to Promote Healthy Aging: Protocol for a Scoping Review
Source: JMIR Res Protoc. 2024 Mar 18;13:e55198. doi: 10.2196/55198 (PMC10985599; doi:10.2196/55198)
Supplement: Multimedia Appendix 1 [file resprot_v13i1e55198_app1.docx]

Example search strategy

| S1 | (MH “Occupational Therapy+”) OR (MH "Occupational Therapy Practice") | 29613 |
| --- | --- | --- |
| S2 | (MH "Occupational Therapists") OR (MH "British Association and College of Occupational Therapists") | 10229 |
| S3 | TI "occupational therap*" OR AB "occupational therap*" | 27365 |
| S4 | S1 OR S2 OR S3 | 47075 |
| S5 | Healthy ageing OR healthy aging OR aging well OR ageing well | 25976 |
| S6 | Active ageing OR active aging | 4292 |
| S7 | Positive ageing OR positive aging | 7457 |
| S8 | Productive ageing OR productive aging | 405 |
| S9 | Successful ageing or successful aging | 3100 |
| S10 | S5 OR S6 OR S7 OR S8 ORS9 | 34647 |
| S11 | S4 AND S10 | 305 |

Note: This search was run on CINAHL Complete (via Ebsco) on 26^th^ September 2023
