# Supplementary material for: Characteristics of Occupational Therapy Interventions to Promote Healthy Aging: Protocol for a Scoping Review
Source: JMIR Res Protoc. 2024 Mar 18;13:e55198. doi: 10.2196/55198 (PMC10985599; doi:10.2196/55198)
Supplement: Multimedia Appendix 2 [file resprot_v13i1e55198_app2.docx]

| **Study ID number** | |  | |
| --- | --- | --- | --- |
| **Reference** | |  | |
| **Date of extraction** | |  | |
| **Empirical (study design)/**  **Non-empirical (type)** | |  | |
| **Country** | |  | |
| **Conceptual framework** | |  | |
| **Intervention name or description** | |  | |
| **Intervention characteristics** (Using the items from TIDieR (Hoffmann et al., 2014) ^##^) |  | | Where located? |
|  | **Brief name** |  |  |
|  | **Why?** |  |  |
|  | **What?** |  |  |
|  | **Procedures** Outcome measures |  |  |
|  | **Who provided?** |  |  |
|  | **How?** |  |  |
|  | **Where?** |  |  |
|  | **When & how much?** |  |  |
|  | **Tailoring** |  |  |
|  | **Modification** |  |  |
|  | **Planned** |  |  |
|  | **Actual** |  |  |
| Impact observed | |  |  |

**#** P or A = page or appendix number of primary paper O= other details

**## Item descriptors**

1. **Brief name =** *Name of phrase that describes the interventions*
2. **Why =** *Rationale, theory, or goal of the elements essential to the intervention*
3. **What =** *Description of any physical of informational materials used in the intervention and how they were provided*
4. **Procedures =** *Description of each procedure, activity, and/or process used in the intervention (including outcome measures)*
5. **Who provided =** *For each provider, describe their expertise, background, and any specific training given*
6. **How =** *Description of modes of delivery*
7. **Where =** *Description of type(s) of location(s) where the intervention occurred*
8. **When and how much =** *Description of the number of times the intervention was delivered and over what period of time*
9. **Tailoring =** *If the intervention was planned to be personalized, titrated, or adapted, then describe what, why, when, and how*
10. **Modifications** = *Description of any changes during the course of the study/ intervention)*
11. **Planned =** *If intervention adherence or fidelity was assessed, describe how and by whom; if any strategies were used to maintain or improve fidelity, describe them.*
12. **Actual =** *If intervention adherence or fidelity was assessed, describe the extent to which the intervention was delivered as planned.*
